# Immunological characterization of the chemically prepared ghosts of *Salmonella Typhimurium* as a vaccine candidate

**DOI:** 10.1186/s12917-021-03112-4

**Published:** 2022-02-18

**Authors:** Sameh Rabea, Aymen S. Yassin, Aly Fahmy Mohammed, Mounir M. Salem-Bekhit, Fars K. Alanazi, Eman Amin Esmail, Nayera A. Moneib, Abd Elgawad M. Hashem

**Affiliations:** 1 Department of Pharmaceutical Sciences, College of Pharmacy, AlMaarefa University, Diriyah 13713 Riyadh, Saudi Arabia; 2 Department of Microbiology and Immunology, Faculty of Pharmacy, Cairo University, Cairo, 11652 Egypt; 3 The International Center for Advanced Researchers (ICTAR-Egypt), Cairo, Egypt; 4 Kayyali Chair for Pharmaceutical Industry, Department of Pharmaceutics, College of Pharmacy, King Saud University, P.O. Box 2457, Riyadh, 11451 Saudi Arabia; 5 Microbiology and Immunology Department, Faculty of Pharmacy, Al-Azhar University, Cairo, 11651 Egypt; 6 The Holding Company for Production of Vaccines, Sera And Drugs (VACSERA), Cairo, Egypt; 7 Faculty of Pharmacy, British University in Egypt (BUE), Cairo, 11837 - P.O. Box 43, Egypt

**Keywords:** Bacterial ghosts, *Salmonella Typhimurium*, Bacterial ghosts’ applications, Vaccination trial, immunological characterization

## Abstract

**Background:**

Bacterial ghosts are the evacuated bacterial cellular membranes from most of the genetic and protein contents which preserved their surface characters. Recently, bacterial ghosts exploited for different biomedical applications, for instance, vaccination. The purpose of this study is to measure the immunogenic protective response of bacterial ghosts of *Salmonella Typhimurium* in animals and to allow future testing this response in humans. The immunologic response was qualitatively, quantitatively, and functionally measured. We have measured the humoral and cellular immune responses, such as immunoglobulins elevation (IgG), increased granulocytes, serum antibacterial activity, clearance of virulence in feces and liver, and the survival rate.

**Results:**

The bacterial ghosts’ vaccine was able to protect 100% of subcutaneously vaccinated rats and 75% of adjuvant subcutaneously vaccinated rats. The lowest survival rate was in the orally vaccinated group (25%). The maximum level of serum IgG titers, as well as serum and feces bactericidal activity (100% eradication), was exhibited in the subcutaneously vaccinated group with adjuvant vaccines followed by the subcutaneously vaccinated one. Additionally, the highest granulocytes’ number was observed in the adjuvant vaccine subcutaneously immunized group. The bacterial load in liver homogenate was eliminated in the subcutaneously vaccinated rats after the virulence challenge.

**Conclusions:**

The bacterial ghosts of *Salmonella enterica* serovar Typhimurium that prepared by Tween 80 Protocol showed an effective vaccine candidate that protected animals, eliminated the virulence in feces and liver. These findings show that chemically induced bacterial ghosts of *Salmonella Typhimurium* can be a promising vaccine.

## Background

The intact unaffected evacuated cellular shells of Gram-negative bacteria that are evacuated from their cellular contents are defined as bacterial ghosts (BGs) [1–6]. According to this definition, Gram-positive bacteria are excluded from the bacterial ghosts. Most of Gram-positive bacteria failed to be lysed by the gene E system. This failure refers to the lack of Gram-positive bacteria of inner and outer membranes that are present in Gram-negative bacteria [4]. However, Gram-positive bacterial ghosts cannot be prepared by the same principle that is used in preparation of Gram-negative ghosts [7].

Recently, the definition of BGs has been extended to include Gram-positive bacteria by applying a new protocol which comprises the use of some chemical agents in their minimum inhibitory concentrations (MIC), and minimum growth concentrations (MGC) and/or some physical factors, like high temperature [7, 8]. Therefore, by using a specific chemical agent in a specific concentration for a certain time, the puncturing of bacterial cells and expelling of the internal contents have been achieved. Although the bacterial cells were pierced, the intactness of the cellular shell was not affected or deformed.

Bacterial ghosts of specific pathogens can be utilized in the preparation of vaccines against these pathogens. However, recently, different approaches have been used to develop human and veterinarian vaccines, such approaches utilize the cellular surface displaying properties (e.g. Protein A in Gram-positive bacteria and specific outer-membrane proteins in Gram-negative bacteria) [9], or advanced molecular techniques [10]. Such techniques involve genetically engineered vaccines, inverted pathogenicity (utilizing virulence factors to prevent or treat a disease), and bacterial ghosts delivery system [11]. The preserved surface structures and components of the BG can induce both innate and adaptive immune responses [12].

The prepared vaccines from bacterial ghosts of several pathogens achieve reasonable protection in animals [11, 13–15]. For example, the bacterial ghosts of *Staphylococcus aureus* that induced by NaOH protected the whole rats’ population against virulent challenge [7]. The survival of the whole rats’ population (100%) against virulent challenge and significant antibodies titer production were achieved by *Listeria monocytogenes* ghosts. The latter ghosts were chemically produced using several reagents, such as calcium carbonate, sodium hydroxide, sodium dodecyl sulphate, and hydrogen peroxide [16]. The prepared *Escherichia coli O157:H7* by E-lysis gene achieved a 93.3% survival rate in rats after the lethal challenge test [17].

In the current study, we have used the chemically induced (tween 80) bacterial ghosts of *Salmonella Typhimurium* in the vaccination of animals to evaluate their immunologic characters. The purpose of the vaccine characterization is to interpret the immunological activity by measuring the ability of the vaccine to provoke responses in form of humoral (e.g., antibodies in serum) and/or cellular (e.g., phagocytosis) immunity. The evaluation of the immune responses includes also the determination of the disappearance of virulence and the survival rate in animals. The study will characterize the safety and efficacy profile of the tested vaccine.

## Materials and methods

### BGs preparation

Bacterial ghosts were produced from *Salmonella enterica* serovar Typhimurium ATCC 11331 using the protocol that was previously described [8]. Briefly, the cells were incubated in Muller-Hinton broth containing 7% v/v tween 80 for 24 h at 37 °C. The grown cells were then exposed to lactic acid (pH = 3.6). By centrifugation, ghosts’ pellets were separated, then washed three times by a sterile solution of half normal saline.

The generation of high-quality ghosts was proved by the visualization of the formed transmembrane tunnels using the scan electron microscope (SEM). The centrifuged pellets of bacterial cells were investigated by SEM. The samples were fixed by glutheraldhyde 2.5% and dehydrated by serial dilutions of ethanol using automatic tissue processor. The samples were dried using CO2 critical point drier (Tousimis Audosamdri-815). The samples were coated by gold sputter coater (SPI-Module). Finally, samples were examined by SEM with amplification power of × 9500 and 20 kV and using high vacuum mode at the Regional Center Mycology and Biotechnology, Cairo, Egypt. Additionally, the quality of produced ghosts was tested by the quantification of released proteins and DNA in the supernatant before washing process using spectrophotometry. The centrifuged pellets of pure cells were stained by Gram stain then visualized by light microscope using amplification power of 1000 x in order to investigate cellular external surface integrity [18].

In order to guarantee a safe vaccine, the pure ghosts’ pellets were tested for viability by subculturing the broth at the end of the incubation period. Then the resultant subcultures viability was tested again by surface streaking on Muller-Hinton agar plates. Finally, the obtained ghosts were lyophilized and stored.

### Experimental animals

The ethical approval for this study was given to us (No. MI 1506) on 28/10/2015 by the Research Ethical Committee, College of Pharmacy, Cairo university to approve this trial. Twenty-four normal young adult male rats (Sprague–Dawley with average weight 150 g) were used in this study and obtained from the animal house of the Faculty of Veterinary Medicine, Cairo University.

### Vaccination of animals

After 2 weeks of acclimatization and housing of the animals by the Holding Company of the Vaccines and Sera, Giza, Egypt, the rats were divided equally into six groups (4 rats in each group). According to previous studies [14, 19], the groups were assigned as the following: group 1, (Control/PBS) was subcutaneously (S.C.) injected by 1 ml PBS. Group 2, (Control/Alum) was S.C. injected by 1 ml adjuvant (Alum). Group 3, (Oral BG) was orally vaccinated by 1 ml suspension of (300 μg/ml) of *Salmonella enterica* serovar Typhimurium ATCC 13311 ghosts (BGs). Group 4, (Oral BG + Alum) was orally vaccinated by equal volumes of BGs + Alum (0.5 ml BG + 0.5 ml alum). Group 5, (S.C. BG) was subcutaneously vaccinated by 1 ml of (300 μg/ml) of BGs suspension. Group 6, (S.C. BG + alum) was subcutaneously vaccinated by equal volumes of BGs + alum (0.5 ml BG + 0.5 ml alum). The vaccination of six groups was repeated every 14 days for 2 cycles.

### Withdrawing blood samples procedure

There are some procedures to withdraw blood samples from animals. The blood samples (2 ml) were withdrawn via retro-orbital sinus puncture [20] from each animal under anesthesia.

### Determination of antibodies response

Serum was separated by centrifugation at 1400 g for 20 min and stored at − 20 °C until analysis. The serum samples were collected every 14 days and were challenged against anti-rat IgG (whole molecule) –Horseradish Peroxidase (HRP) conjugate antibodies followed by ELISA analysis. As described before [11], the live *Salmonella* cells were added to each well of the 96- microwell ELISA plates as 50 μl (10 × 10^9^) and left to dry. Plates were blocked with 10% bovine serum albumin (BSA) for 1 h. Sera samples were 2-fold serially diluted and dispensed to the plates for 1 h, then were incubated and washed with the buffer (200 μl). The anti-rat IgG HRP conjugate was added to the whole plates as 1/1000. The plates were incubated for 1 h then washed as previously described. Then, substrate buffer was added (50 μl). The developed color was stopped using 2 N H_2_SO_4_. The readings were taken using the microplate reader at 450 nm filter. Finally, the mean optical density was plotted against the time post vaccination.

### Antibacterial activity in serum

After 42 days and finishing of the whole vaccination program, serum (25 μl) was collected from each group and challenged by addition of 100 μl (1.5 X 10^8^ CFU/ml) suspension of live *Salmonella enterica* serovar Typhimurium ATCC 13311. The mixture \was incubated for 1 h at 37 °C. Then it was mixed uniformly with Shigella-Salmonella (S-S) media to count viable cells [19].

### The granulocytes’ percentage calculation

Forty-two days later, 2 ml of withdrawn blood (from each group) were examined for differential complete blood count (CBC). The differential CBC performed on an automated blood analyzer then the granulocytes’ percentage was calculated, and abnormal results were reported.

### Virulence challenge-antibacterial response in feces

After finishing of the whole vaccination program, every rat was infected by S.C. injection of 1 ml (1.5 X 10^8^ CFU/ml) live *Salmonella enterica* serovar Typhimurium ATCC 13311 cells. After 1 week, feces samples (1 g) were collected from each group and were mixed uniformly with S-S media to investigate bacterial counts [21].

### Virulence challenge-antibacterial response in liver homogenate

One week after infection, all the rats were sacrificed and liver tissues (1 g) were homogenized and mixed uniformly with S-S media to investigate viable bacterial counts [21].

### Termination of the animals

After finishing all required procedures, all animals were terminated by decapitation under anesthesia. Their cadavers and tissues were frozen until incinerated according to the standard procedure [22].

### Statistical analysis

The significant difference between the means at a confidence interval of 95% were compared using ANOVA for parametric data. The significant difference was determined while comparing the released quantities of proteins and DNA, the titers of antibodies, and CFU (in feces, serum, and liver homogenate) that resulted among different experimental groups. The survival rate using Kaplan-Meier- curves were generated. *P* values < 0.05 and *P* values < 0.0001 were considered statistically significant. All the previous statistical analyses were accomplished using GraphPad Prism program version.6.01.

## Results

### Production of BGs

**Fig. 1:**
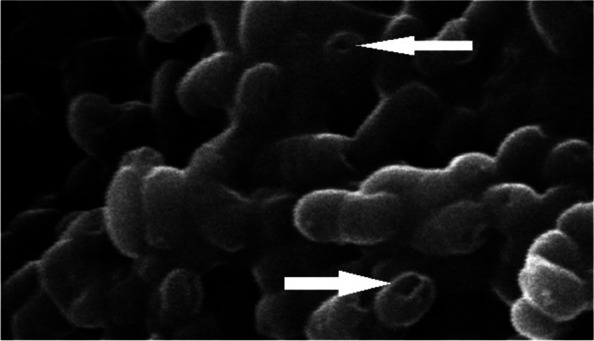
Scanning electron micrograph showing the perforating effect of tween 80 on the cellular membrane of *Salmonella’s* cells (amplification power of × 9500 and 20 kV). The arrows are indicating surface pores

**Fig. 2:**
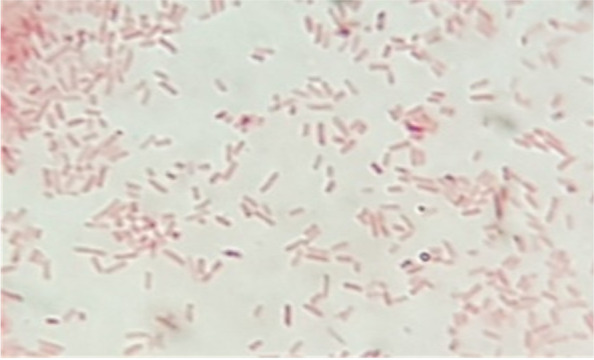
Light micrograph revealing surface integrated and unaffected *Salmonella* Gram- stained cells (amplification power of 1000 x)

The obtained ghosts were intact bacterial shells showing several intra-membranous tunnels that obviously were shown in the SEM micrograph (Fig. 1). The optimizing physical and chemical conditions of the incubation led to the production of high-quality *S. typhimurium* ghosts. The high-quality of ghosts was proved by the release of high amount of proteins and DNA which are, 2975 μg/ml and 786 μg/ml respectively. In comparison to untreated cells, the released proteins and DNA were almost null which indicated a significant difference (*P* value < 0.05 ANOVA analysis). The light microscopic examination revealed intact stained bacilli as shown in the light microscope micrograph (Fig. 2). The generated ghosts are considered safe as the subculture of prepared ghosts gave negative growth and zero viable cells.

### Determination of antibodies response

**Fig. 3:**
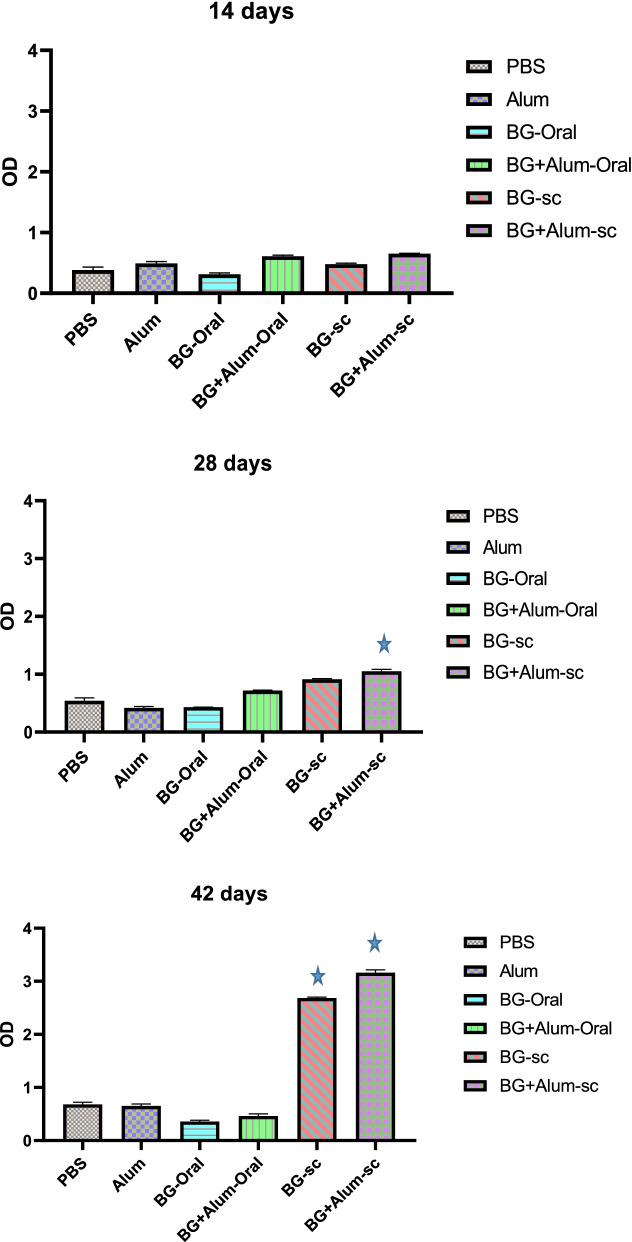
The OD of IgG titers produced versus days among different vaccination groups. *P* value 0.0001 vs PBS, Alum, and BG-Oral groups after 28 days and vs PBS, Alum, BG-Oral, and BG-Alum-Oral groups after 42 days

At the end of the first vaccination cycle (14 days), there was no significant difference in the quantities of serum IgG’s among all the six groups (Control/PBS, control/alum, Oral BG, Oral BG + alum, S.C. BG, and S.C. BG + alum). At the end of the second cycle (28 days), the highest IgG titer was shown in the subcutaneously vaccinated group (S.C. BG + alum), with significant difference *P* value 0.0001(one-way ANOVA), Fig. 3.

Finally, at the end of the last cycle of vaccination (42 days), both subcutaneously vaccinated groups (S.C.BG and S.C. BG + alum) showed a significant difference *P* value 0.0001 (one-way ANOVA) in the serum IgG antibodies quantity. The subcutaneously vaccinated group (S.C.BG + alum) showed the ultimate highest serum IgG during the whole 42-day vaccination period which reached 3.125 optical density (OD). The subcutaneously vaccinated group by BG (S.C. BG) was hitting 2.686 OD at 450 nm, Fig. 3.

### Antibacterial activity in serum

**Fig. 4:**
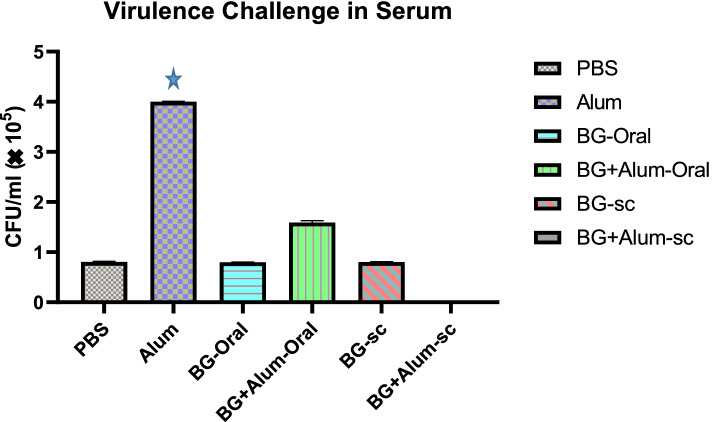
The viable count of *Salmonella* in rats’ serum of different vaccinated groups. *P* value 0.0001 vs PBS, BG-Oral, BG-sc, and BG-Alum-sc groups

The collected sera from all groups were challenged against live *Salmonella* cells. The virulence challenge test showed the failure of all vaccinated groups except the subcutaneously vaccinated group by BG with adjuvant (S.C. BG + alum) showing full protection. The sera of this group showed absence of any viable *salmonella* cells, Fig. 3. Other groups showed high viable cell counts, Fig. 4.

### The percentage of granulocytes

**Fig. 5:**
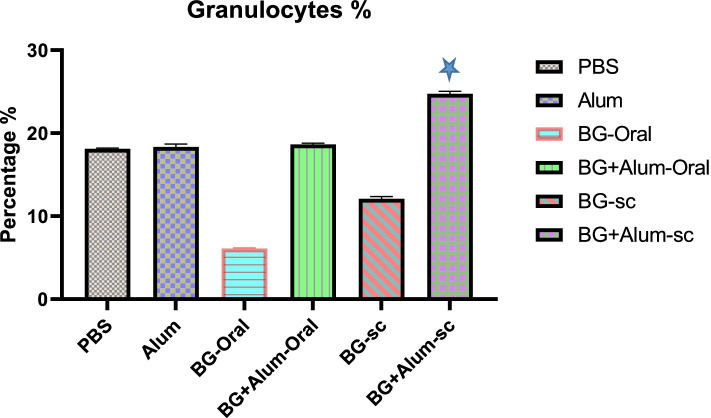
The percentage of granulocytes among different vaccinated groups. *P* value 0.0001 vs BG-Oral group

The percentage of granulocytes was significantly increased *P*-value 0.0001 (one-way ANOVA) among the subcuteneously vaccinated group by BG combined with alum (S.C. BG + alum). The granulocytes were present in almost the same pecentage (18%) among other groups, Fig. 4. The least percentage (6.2%) of granulocytes were shown among the orally vaccinated group by BG (Oral BG). The subcuteneously vaccinated group by BG showed only granulocytes’ percentage of 12%, Fig. 5.

### Virulence challenge-antibacterial response in feces

**Fig. 6:**
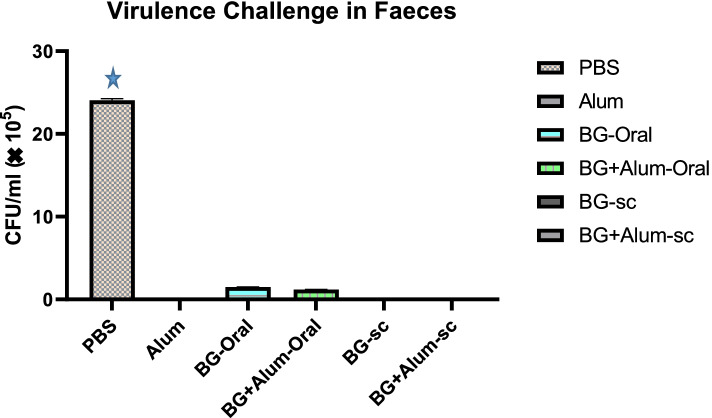
The viable count of *Salmonella* in rats’ feces among different vaccinated groups. *P* value 0.0001 vs all groups

One week later after the whole vaccination period up, all groups were subcutaneously infected by a fresh standard culture of *Salmonella enterica* serovar Typhimurium ATCC 11331. One week after the infection, the fecal bacterial count had been completed**.** The maximum antibacterial activity response was shown among the subcutaneously vaccinated groups by BG and BG + alum, as well as, the alum control group (S.C. BG; S.C. BG + alum; Control/alum). All the previously mentioned groups showed the disappearance of viable *Salmonella* cells in the feces, Fig. 6.

Both orally vaccinated groups (Oral. BG and Oral BG + alum) along with the Control/PBS group failed to be protected against the virulent bacteria, as is shown in the feces variable viable cells counts. The highest viable counts (2.4 10 ^6^ CFU/gm) were shown among the PBS control group, while the orally vaccinated groups by BG and BG + alum showed 1.5 10^5^ and 1.16 10^5^ CFU/gm respectively *P* value 0.0001 (One-way ANOVA), Fig. 6.

### Virulence challenge-antibacterial response in liver homogenate

None of the vaccinated group have shown any liver protection against the virulence challenge by *salmonella* except the subcutaneously vaccinated group by BG with adjuvant (S.C. BG + alum). The other vaccinated groups have shown variable viable cells counts. The subcutaneously vaccinated group by BG (S.C. BG) have shown significant decrease in the virulent viable count *P*-value 0.0001 (One-way ANOVA) which was 7000 CFU/gm. Both control groups, PBS and alum, have shown an uncountable number of viable counts, Table 1.

**Table 1:** The viable count of *Salmonella* in rats’ liver homogonate among different vaccinated groups

Vaccine type	Viable count CFU/gm
PBS	uncountable
BG Oral	1.3 10 ^5^
BG + Alum Oral	3 10 ^4^
BG SC	7000
Alum	uncountable
BG + Alum SC	0

*P* value 0.0001 vs all groups

### The survival animals after virulence challenge test

**Fig. 7:**
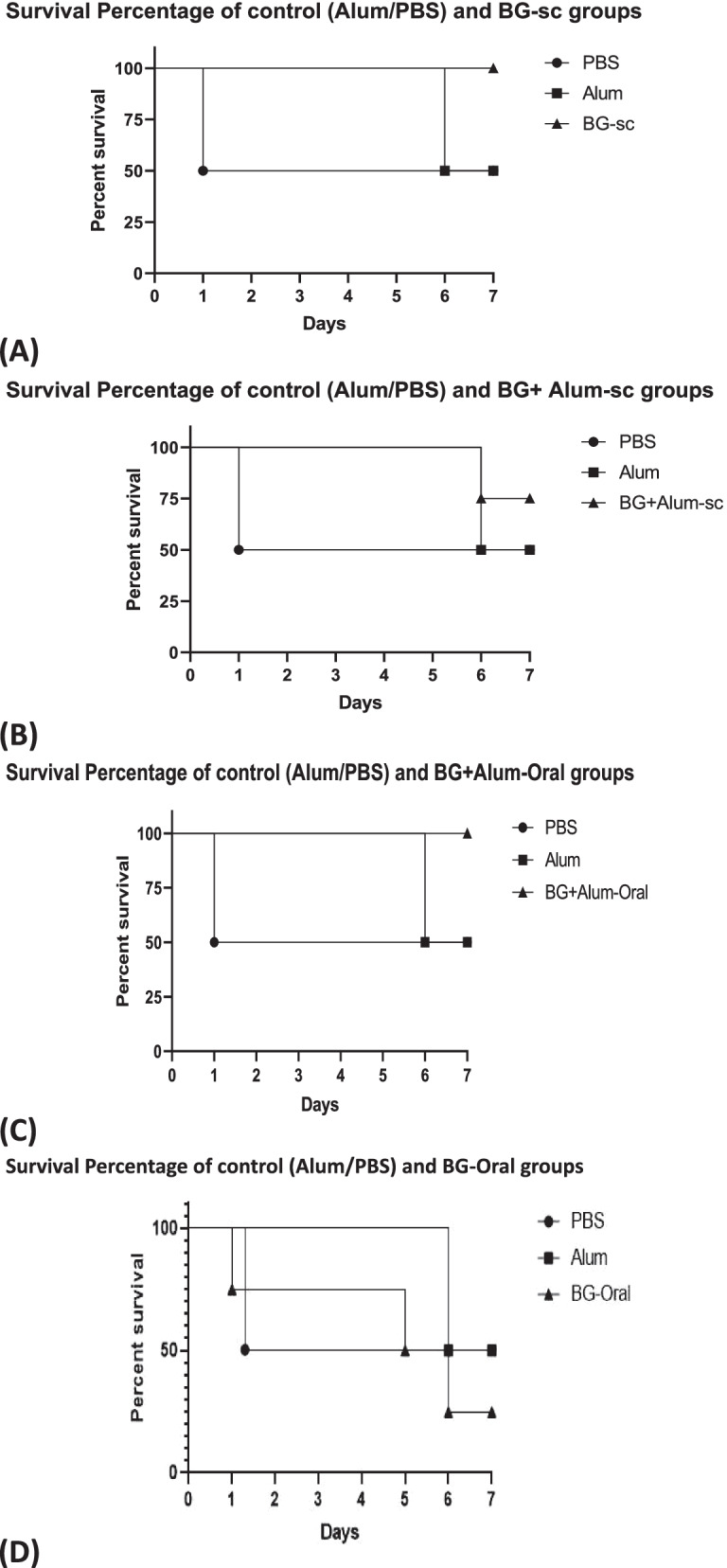
The survival percentage among all vaccinated groups

After 7 days of virulence challenge test and intentional infection by *salmonella* to all vaccinated groups, full survival was achieved among both groups of subcutaneously vaccinated groups (S.C. BG) and orally vaccinated group by BG an adjuvant (Oral BG + alum)). Only one rat (25%) died among the group that subcutaneously vaccinated by BG with adjuvant (S.C. BG + alum). The survival percentage was the same (50%) in both control groups (Control/PBS and Control/alum). The lowest survival percentage (25%) was among the orally vaccinated group by BG (Oral BG), Fig. 7.

## Discussion

Recently, different approaches have been used to develop human and veterinarian vaccines. Such approaches either utilize the cellular surface displaying properties (e.g., Protein A in Gram-positive bacteria and specific outer-membrane proteins in Gram-negative bacteria) [23], or advanced molecular techniques. Such techniques involve genetically engineered vaccines, inverted pathogenicity (utilizing virulence factors to prevent or treat a disease) [11], and bacterial ghosts’ delivery system [11, 13]. The preserved surface structures and components of the BG can induce both innate and adaptive immune response [24].

Using of bacterial ghosts is one of promising approaches to obtain a competent vaccine [25]. The need for an easy, economic, and feasible method for preparation of BGs is simultaneously amplified with the increased BGs’ applications. Traditionally, BGs were prepared by genetic means utilizing the E-lysis gene which is exclusively effective by Gram-negative bacteria in the production of a well-formed transmembrane tunnel [2]. Multiple steps used to be applied to obtain a hundred percent (100%) of non-living lysed cells [26]. The high cost and sophistication are limiting factors of using the genetic methods for the preparation of bacterial ghosts. Alternatively, Chemical agents can be used in critical concentrations and specific periods of time for preparation of Gram positive BGs [7, 27], Gram negative BGs [12, 15, 21], yeasts’ ghosts [28], and even viral ghosts [29].

In the current study, the highest serum bactericidal activity (100% eradication) was achieved in the BG+ alum subcutaneously immunized animals. *Salmonella* cells disappeared from the feces of the immunized rats by subcutaneous injection of both BG and BG + alum vaccine after virulence challenge test. In previous studies, the minimum inhibitory concentration (MIC) of sodium hydroxide induced BG of *S. aureus* showed a significant lowering of the total bacterial load within the internal organs (liver, spleen, lungs, and kidneys) of all vaccinated rats’ groups (orally, subcutaneously, and intravenously) [12].

In a related study, *Salmonella enteritidis* ghosts (SEG) that were also prepared by the same agent (NaOH) showed a comparable immune response. The highest serum bactericidal effect was shown in the intramuscular (SEG) with complete Freund’s adjuvant followed by the orally vaccinated rats’ group at the sixth week. Similarly, the intramuscular (SEG) vaccine with adjuvant gave the highest IgG titers and showed the least bacterial load in the internal organs’ homogenates, followed by intramuscular, then orally vaccinated group in week 8 and week 10. All vaccinated groups exhibited significant humoral and cellular immune responses in comparison to the non-vaccinated rat groups [15].

In this study, the highest IgG titers were at the maximum level in the subcutaneously vaccinated group with BG + alum followed by the BG subcutaneously vaccinated group on the last day of immunization program. The bacterial load in the liver homogenate significantly reduced in the subcutaneously vaccinated rats by BG only after virulence challenge and disappeared in the vaccinated group by BG + alum. In a previous study, the highest IgG antibody activity and the serum bactericidal activity were elicited in the subcutaneously vaccinated group at week 9, followed by intravenously, then finally, orally vaccinated group. Likewise, CD4+ and CD8+ T-cell populations were produced in the largest percentage in the subcutaneously, intravenously, then orally vaccinated groups. The survival rate was 100% in the intravenously immunized, while about 60% in the non-immunized group [7].

In another trial, the immunized cattle by genetically prepared BGs of *Brucella suis* S2 showed the same titers of IgG, interleukin 4, INF-γ, and T-cells as is shown in the conventional (formalin-killed Brucella) immunized cattle [30].

In the current trial, the survival rate in the BG subcutaneously vaccinated rats’ group was 100%. This was followed by the BG-adjuvant subcutaneously vaccinated group (75%), while the lowest survival rate was in the orally vaccinated group. In a corresponding results in another study, the immunization of rats by subcutaneous injection of BGs of *Listeria monocytogenes* that were prepared by sponge-like protocol (a chemically induced method) [31] that protected the immunized rats by 100% in the opposite of 0% percent survival in non-immunized animals. By using the same protocol, sponge-like protocol, *Salmonella Typhimurium* ATCC 14028 was turned into BGs then orally administered. The serum of the orally vaccinated group showed agglutination reactions between antigen O and H against antibodies that prove the correct *Salmonella* envelop structure that may protect against live *Salmonella* infection [14].

In a previous study, the prepared *Klebsiella pneumonia* by the same protocol (Sponge-like chemical protocol) gave both cellular and humoral immune responses in form of subcutaneous (the highest activity), inhalation, intraperitoneal, and intramuscular routes.

In contrast to the result of the current study, the oral vaccines of *salmonella*’s BGs failed to protect rats. However, another study showed that orally vaccinated animals by BGs of *E. coli* O157:H7 [17] and *H. pylori* [13] were survived by 93 and 100% respectively. Additionally, the bacterial colonization in the intestine (*E. coli* BG vaccine) and stomach (*H. pylori* BG vaccine) was reduced. Also, there was significant existence of anti *H. pylori* and Omp-specific antibodies in *H. pylori* BG vaccinated animal group [13].

In this study, the highest percentage of granulocytes was raised in the BG + alum subcutaneously immunized group. Significant induction of dendritic cells antigen presentation and release of different interleukins and anaphlytoxins were followed the subcutaneous immunization of rabbits by BG of *V. cholerae* H1 strains [32]. It was compatible with the results of the current study that 100% protection was accomplished by the immunization of both rabbits and mice by genetically induced *Pasturella multocida* and *Pasturella haemolytica* BGs by subcutaneous route [33].

The chemically prepared (tween 80 protocol) *Salmonella enterica* serovar Typhimurium BGs vaccines were able to protect all the immunized rats (survival rate 100%) without adjuvant. It also offered both humoral and cellular immune responses in the case of the subcutaneous route of administration. The minor immune response of the oral BG vaccine may refer to the gastric intestinal digestion as well as possible intentional reflux by the animal itself.

## Conclusions

The immunological characters of *salmonella* BG that have been revealed in this study show that BGs can be a promising platform for effective vaccine production to immunize against a variety of bacterial infections in animals. However, further preclinical trials are required to assure the ghosts’ vaccines’ safety and applicability, before shifting to the phases of the clinical trials can be initiated.

## Data Availability

Not applicable.

## Abbreviations

ANOVA: Analysis of variance
ATCC: The American type culture collection
APC: Antigen presenting cells
BG: Bacterial ghosts
CBC: Complete blood count
CD4+: Cluster of differentiation 4+
CD8+: Cluster of differentiation 8+
CFU: Colony forming unit
DC: Dendritic cells
ELISA: Enzyme linked immunosorbent assays
HPBG: *Helicobacter pylori* ghost
HPLC: High-performance liquid chromatography
IFN-γ: Interferon-γ
IL-: Interleukin-
IM: Inner membrane
KPG: *Klebsiella pneumoniae* ghost
LMG: *Listeria monocytogenes* ghost
LPS: Lipopolysaccharide
MGC: Minimum growth concentrations
MHC: Major histocompatibility complex
MIC: Minimum inhibitory concentration
MOMP: Major outer membrane protein
OM: Outer membrane
Omp: Outer membrane protein
PAMP: Pathogen associated molecular patterns
PBS: Phosphate buffered saline
SEG: *Salmonella enteritidis* ghost
SEM: Scanning Electron Microscope
SLRP: Sponge-like reduced protocol
TA: Target antigen
Th: Thymocyte-helper cells
TLR: Toll-like receptor
VCG: *Vibrio cholerae* ghost
VPG: *Vibrio parahaemolyticus* ghost
